# Gendered trends in unpaid labour by educational level in Sweden 2004–2022

**DOI:** 10.1186/s12889-026-29473-9

**Published:** 2026-09-16

**Authors:** Anu Molarius, Fredrik Granström, Jennifer Ervin

**Affiliations:** 1 Centre for Clinical Research, Region Värmland, S-651 85 Karlstad, Sweden; 2 Department of Public Health Sciences, Karlstad University, Karlstad, Sweden; 3 Department of Public Health, Region Värmland, Karlstad, Sweden; 4 Centre for Health Policy, Melbourne School of Population and Global Health, The University of Melbourne, Parkville, Australia; 5 Social Equity Research Centre, Global Urban Social Studies, RMIT University City Campus, Melbourne, Australia

**Keywords:** Unpaid labour, Domestic work, Education, Social determinants of health, Gender, Time trends

## Abstract

**Background:**

Unpaid labour has been recognised as an important yet neglected social determinant of health. We investigated gendered trends in unpaid labour/domestic work by educational level in prime working-age Swedish adults (30–49 years) from 2004–2022.

**Methods:**

The study is based on a postal survey sent to a random general population sample of adults from central Sweden in 2004, 2008, 2012, 2017 and 2022. The 30–49-year age group included between 5,500 and 12,200 respondents depending on the survey year. Trends in hours spent in domestic work and in experiencing domestic work as burdensome by gender and educational level were estimated by age-standardised proportions with corresponding 95% confidence intervals.

**Results:**

Women reported spending more time in domestic work than men throughout every survey period from 2004 to 2022. Over time, hours spent in domestic work decreased in women whereas no trend was observed in men. In 2022, 31% of women spent more than 20 h per week in domestic work compared to 20% of men. Among women, educational level appeared to make no difference to prevalence of high domestic work time whereas, among men, it was consistently greater among those with higher educational levels. In general, women experienced domestic work as burdensome to a higher degree than men did.

**Conclusion:**

As gender differences in unpaid labour persist in Sweden, more effort is needed to implement social policies to increase men’s participation in domestic work, especially among men with low and middle educational levels, which has the potential to reduce gendered health inequities.

**Supplementary Information:**

The online version contains supplementary material available at https://doi.org/10.1186/s12889-026-29473-9.

## Introduction

Unpaid labour has been recognised as an important yet neglected social determinant of health [1]. Unlike paid employment, scant research attention has been directed towards unpaid labour despite it being a ubiquitous requirement of human day-to-day living, consuming billions of hours of time each day across the globe [2, 3]. Importantly, unpaid labour is highly gendered, with women performing three-quarters of the world’s unpaid labour burden [2, 4]. Crucially, high unpaid labour demands not only have paid work force and economic implications but can also impact mental health and wellbeing [3, 5–7], self-rated health [8, 9] and sickness absence [10, 11].

There are, however, socioeconomic differences in men’s and women’s contribution to unpaid work [12]. Notably, higher educational levels have been recognised as being closely associated with larger contributions to unpaid labour (including childcare) among men [13]. Moreover, observed socioeconomic differences in mental health [14] may be linked to socioeconomic differences in unpaid labour [15]. Therefore, understanding gender and socioeconomic differences in unpaid labour trends is valuable and much needed.

Whilst no universal definition for unpaid labour exists, it is typically understood to be broadly inclusive of all duties and responsibilities, inclusive of care, performed to maintain a household and its members without financial compensation [3, 16]. Multiple terms are used to denote unpaid labour across academic, political, and methodological realms including unpaid work, unpaid care work, domestic work/labour, and housework/household work/labour [3]. However, not all terms are synonymous given some measures of unpaid labour are inclusive of unpaid caregiving (coined ‘total’ unpaid labour), whilst others pertain solely to household chores (housework) [3]. In the context of this study, unpaid labour and domestic work are used interchangeably (and represent ‘total’ unpaid labour).

In Sweden, men spend more time in unpaid labour than men in most other countries [17]. Indeed, the Nordic countries have long been celebrated for leading the way in promoting more gender egalitarian societies. Nonetheless, substantial inequities in the division of labour persist in Scandinavia [17]. Whilst the Swedish government has a specific policy target for “an even distribution of unpaid housework and care work” [18], Swedish women still spend considerably more time doing unpaid work than men [5, 17]. Importantly, these inequities persist despite women’s long-standing equal labour force participation in Sweden. There have been minimal differences in employment rates between men and women since the beginning of 2000’s (around 75% in men vs around 70% in women in the age group 15–64 years) [19], albeit more women work part-time than men [20].

Importantly, combining paid work with high unpaid labour demands results in a double burden effect [3] (also coined the second shift [21]), which is theorised to lead to stress, role overload, role conflict and time poverty – all of which may negatively impact health and wellbeing [22–25]. Moreover, it follows that unpaid labour can be experienced as burdensome via these same pathways but could also be attributable to many unpaid labour tasks (such as housework) being perceived as a collection of uninteresting, unappreciated, and unpleasant tasks [23, 26].

To date, and to the best of our knowledge, only a handful of studies have examined the influence of education on gendered time patterns and unpaid labour/domestic work. A cross-sectional study from 2009, using data from the Netherlands, Sweden, and the US, reported that less educated women in all three nations spent more time in housework than highly educated women [27]. The findings for men were more varied, with clear educational level differences evident only for Swedish men, with low-educated men spending the least time in housework. Moreover, a 2021 paper examined the cross-sectional association between women’s educational levels and housework participation across cultural contexts (Japan, Taiwan, and the US) and found that single women with higher levels of education spend less time on housework than women with less education in all contexts [28]. Pertinent to the current study, some research investigating trends over time has been conducted. A 2019 Japanese study examined educational attainment and domestic work contribution trends between 1991 and 2016, reporting an overall shift in higher unpaid labour time for more educated Japanese men [29]. Similarly, a 2014 European study (using cross-national time use data) demonstrated more egalitarian division of labour (increases in contribution to childcare and core domestic work) among more highly educated fathers [12].

The aim of this study is to investigate the gendered trends in unpaid labour/domestic work by educational level in a prime working-age sample of Swedish adults (30–49 years) from 2004 to 2022. Based on the premise that higher levels of education are associated with more egalitarian attitudes and behaviours, we hypothesise that higher educational attainment will be associated with increased contribution to unpaid labour (in time spent and in experiencing it as burdensome), especially for men, and that the gender difference in domestic work has decreased over the time period.

## Methods

### Data source and sample

This study is based on cross-sectional postal surveys (Life and health) sent to a random population sample aged 18–84 years in 2004, 2008, 2012, 2017 and 2022 (18 years or older in 2017 and 2022), with the aim to monitor living conditions, lifestyle factors and health in the general population. The study area includes 55 municipalities with over 1 million inhabitants in five counties (Sörmland, Uppsala, Värmland, Västmanland and Örebro, except for 2012 when Värmland county did not participate) in Mid-Sweden. The sampling frame was the population register at Statistics Sweden, the statistical administrative authority in Sweden, covering all inhabitants of the study area. Data collection was discontinued after two postal reminders. In 2022, up to three short message service (SMS) reminders were also sent to persons with a registered mobile phone number. The overall response rates for each survey year were 64%, 59%, 51%, 44% and 45%, respectively. More information about the surveys and questionnaire used can be found elsewhere [5, 8, 30].

Individuals in the sample were informed that questionnaires would be linked to the Swedish official registries via personal identification numbers, providing information on gender, age, and educational level. Respondents thus accepted the linking of registry data by informed consent. Personal identification numbers were deleted directly after record linkage. Statistics Sweden carried out the sampling, collected the data, performed the linkage with register data and delivered de-identified data to the county councils/regions.

For the current study, our main analytic sample was restricted to adults aged 30–49 years. This age group was selected given it captures both prime working years as well as key child rearing years when unpaid labour demands are typically the highest in households and since those in prime working age groups spend approximately double the amount of time in domestic work compared to other age groups [8]. The resultant study population for each survey year was 12,148, 10,493, 7,173, 5,564 and 6,312 respondents, respectively. For completeness, we also conducted corresponding analyses for the older age group, 50–69 years, for which further details and results are reported in our supplementary file [see Supplementary file 1].

### Measures

#### Unpaid labour

At each survey year, there were two questions pertaining to unpaid labour/domestic work. The first one was: “How many hours a week on average do you spend working at home that is not paid work? e.g. taking care of children, nursing relatives, buying groceries, cooking, paying the bills, washing the laundry, cleaning, taking care of a car, a house or a garden”. The categorical answer options were: 0–2, 3–10, 11–20, 21–30, and 31 or more hours per week. The measure was dichotomised into up to 20 h/week or greater than 20 h/week for the trend analyses by educational level. The cut-off value of 20 h/week aligns with previous studies [5, 8, 31] and corresponds to a half-time working week.

The second question asked how often the respondent experienced domestic work as burdensome (never, seldom, sometimes, most of the time, all the time). For our study these alternatives were condensed into three categories: “never/seldom”, “sometimes” and “all or most of the time”—with the last category (domestic work being burdensome all or most of the time) represented in the trend analyses by educational level.

### Educational level

Educational level data was obtained from a national education register and categorised into three levels: low (elementary school), medium (upper secondary school), and high (at least 3 years of university or corresponding education).

### Other

Gender and age of respondents were based on official registers. Household structure data was gleaned from the surveys. Whether respondents had children under 18 years living at home was defined from the question: “With whom do you share a home? That is, who do you live with during most of the week.”. Those who answered “Children younger than 18 years” were coded as yes, otherwise the variable was coded as no. This information was only available for survey years 2017 and 2022.

### Statistical methods

Trends from 2004 to 2022 in hours spent in domestic work and in experiencing domestic work as burdensome, including comparisons across educational levels, were estimated by age-standardised proportions with corresponding 95% confidence intervals. All analyses were stratified by gender. The statistical program SPSS, version 28.0, was used and age-standardisation was calculated by 10-year age groups.

### Ethical considerations

At the time of the data collection in years 2004 and 2008, the Ethical Review Act of Sweden (2003:460) did not require ethics approval given the anonymity of the data. In 2012 and 2017, the survey was approved by The Regional Ethical Review Board of Uppsala (EPN 2012/256, and EPN 2015/417). In 2022, an authorisation from the Swedish Ethical Review Authority was obtained (Dnr 2021–05814-01). The data is protected according to the Public and Privacy Act (2009:400, Chapter 24, Sect. 8).

## Results

Table 1 presents the sample characteristics by year. Somewhat more women than men answered the questionnaire in all years. More participants were in the 40–49-year age group than the 30–39-year group. The proportion of participants with low educational level decreased from 10% in 2004 to 5% in 2022, while the proportion with high educational level increased from 35 to 56%. About two thirds of the participants had children under 18 years living at home.

**Table 1:** Characteristics of the study population by year

Year	2004	2008	2012	2017	2022
**N**	12 148	10 493	7 173	5 564	6 312
**Gender (%)**					
Women	56.7	57.8	58.6	58.3	59.3
Men	43.3	42.2	41.4	41.7	40.7
**Age group (%)**					
30–39 years	49.3	46.1	41.8	42.0	43.9
40–49 years	50.7	53.9	58.2	58.0	56.1
**Educational level (%)**					
Low	10.4	8.5	7.4	4.9	5.4
Middle	54.4	53.3	46.5	41.8	38.3
High	35.2	38.1	46.0	53.3	56.3
**Children under 18 years (%)**					
Yes	-	-	-	66.9	65.9
No	-	-	-	33.1	34.1

In general, women spent more time in domestic work than men during every study period from 2004 to 2022 (p < 0.05). For example, the prevalence of spending ≥ 31 h/week in domestic work was consistently about double among women compared to men (Table 2). However, among women, total time spent in domestic work decreased over the survey years (p < 0.05). The proportion spending ≥ 31 h or more per week decreased from 25 to 18%, and the proportion spending 21–30 h/week from 21 to 15%. No change was seen among men. The proportion who sometimes experienced domestic work as burdensome decreased among women. However, the proportion who experienced domestic work all or most of the time as burdensome was about 14% in women and about 8% in men and remained stable in both men and women from 2004 to 2022.

**Table 2:** Age-standardised distribution of hours spent in domestic work and burdensome domestic work among women and men aged 30–49 years by year

Year		2004 % (95% CI)^1^	2008 % (95% CI)^1^	2012 % (95% CI)^1^	2017 % (95% CI)^1^	2022 % (95% CI)^1^	Difference 2004–2022^2^
**Women**							
**Time spent in domestic work (%)**							
0–2 h/week	2.3 (2.0, 2.7)		2.6 (2.2, 3.0)	3.2 (2.7, 3.8)	4.3 (3.7, 5.0)	5.6 (4.8, 6.3)	3.2
3–10 h/week	23.8 (22.6, 24.9)		27.6 (26.3, 29.0)	35.8 (34.1, 37.6)	29.7 (27.8, 31.5)	35.8 (33.9, 37.7)	12.0
11–20 h/week	29.1 (27.8, 30.4)		30.6 (29.2, 32.0)	30.1 (28.4, 31.7)	30.8 (28.9, 32.7)	27.4 (25.8, 29.1)	−1.7
21–30 h/week	20.8 (19.7, 21.9)		18.6 (17.6, 19.7)	15.0 (13.8, 16.2)	17.7 (16.3, 19.2)	14.6 (13.4, 15.8)	−6.2
31 + h/week	24.0 (22.8, 25.1)		20.5 (19.3, 21.7)	15.9 (14.6, 17.1)	17.5 (16.0, 19.0)	16.7 (15.3, 18.0)	−7.3
**Experiences domestic work as burdensome (%)**							
Never/seldom	26.5 (25.3, 27.7)		27.8 (26.5, 29.1)	30.6 (28.9, 32.3)	31.1 (29.2, 33.1)	31.0 (29.3, 32.8)	4.6
Sometimes	59.8 (58.0, 61.6)		58.3 (56.4, 60.2)	56.5 (54.3, 58.8)	54.8 (52.3, 57.4)	53.9 (51.6, 56.3)	−5.9
All or most of the time	13.7 (12.8, 14.6)		13.9 (13.0, 14.9)	12.9 (11.8, 14.0)	14.1 (12.8, 15.4)	15.0 (13.7, 16.2)	1.3
**Men**							
**Time spent in domestic work (%)**							
0–2 h/week	9.2 (8.4, 10.0)		9.5 (8.6, 10.4)	8.9 (7.9, 10.0)	8.3 (7.1, 9.5)	10.4 (9.2, 11.7)	1.3
3–10 h/week	41.8 (40.1, 43.6)		44.3 (42.3, 46.2)	44.3 (42.0, 46.7)	40.4 (37.9, 43.0)	42.3 (39.8, 44.9)	0.5
11–20 h/week	28.3 (26.9, 29.8)		26.6 (25.1, 28.1)	27.9 (26.0, 29.7)	28.4 (26.2, 30.5)	26.8 (24.8, 28.8)	−1.5
21–30 h/week	11.8 (10.8, 12.7)		11.5 (10.4, 12.5)	11.6 (10.3, 12.8)	12.3 (10.9, 13.8)	11.9 (10.6, 13.3)	0.2
31 + h/week	9.0 (8.2, 9.8)		8.1 (7.3, 9.0)	7.2 (6.2, 8.2)	10.5 (9.1, 11.9)	8.6 (7.4, 9.7)	−0.4
**Experiences domestic work as burdensome (%)**							
Never/seldom	45.7 (43.9, 47.5)		48.7 (46.6, 50.7)	45.8 (43.4, 48.2)	48.6 (45.8, 51.4)	46.3 (43.7, 48.9)	0.6
Sometimes	45.9 (44.0, 47.7)		43.4 (41.5, 45.3)	46.4 (43.9, 48.8)	43.4 (40.8, 46.1)	44.3 (41.7, 46.9)	−1.6
All or most of the time	8.4 (7.6, 9.2)		7.9 (7.0, 8.7)	7.7 (6.7, 8.8)	8.0 (6.8, 9.1)	9.5 (8.3, 10.7)	1.1

^1^ *CI* Confidence interval

^2^ Difference 2004–2022 is regarded as statistically significant with p-value < 0.05 when the 95% CIs for 2004 and 2022 do not overlap

**Fig. 1:**
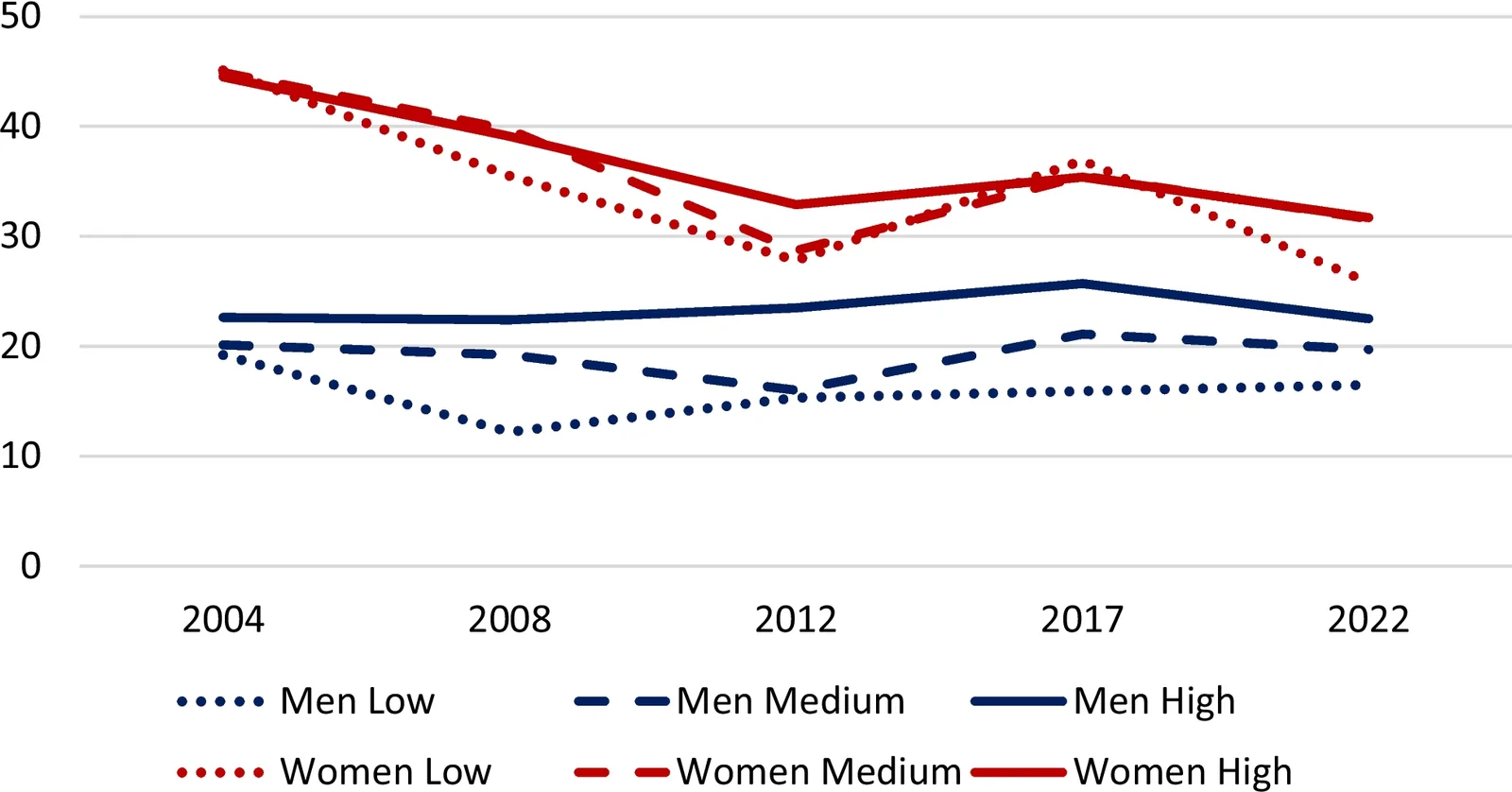
Age-standardised prevalence (%) of spending more than 20 h per week in domestic work among women and men aged 30–49 years by level of education and year

Figure 1 shows the age-standardised prevalence of spending > 20 h/week in domestic work by gender, educational level, and year. Among women, there was no difference in the prevalence of spending > 20 h/week in domestic work between educational levels, except in 2022 when the prevalence was lowest among those with low educational level (*p* < 0.05). Among men, a consistent difference between educational levels was observed. The prevalence of spending > 20 h/week in domestic work was greatest among those with high educational level and lowest among those with low educational level (*p* < 0.05 for difference between high and low educational levels in all years).

**Fig. 2:**
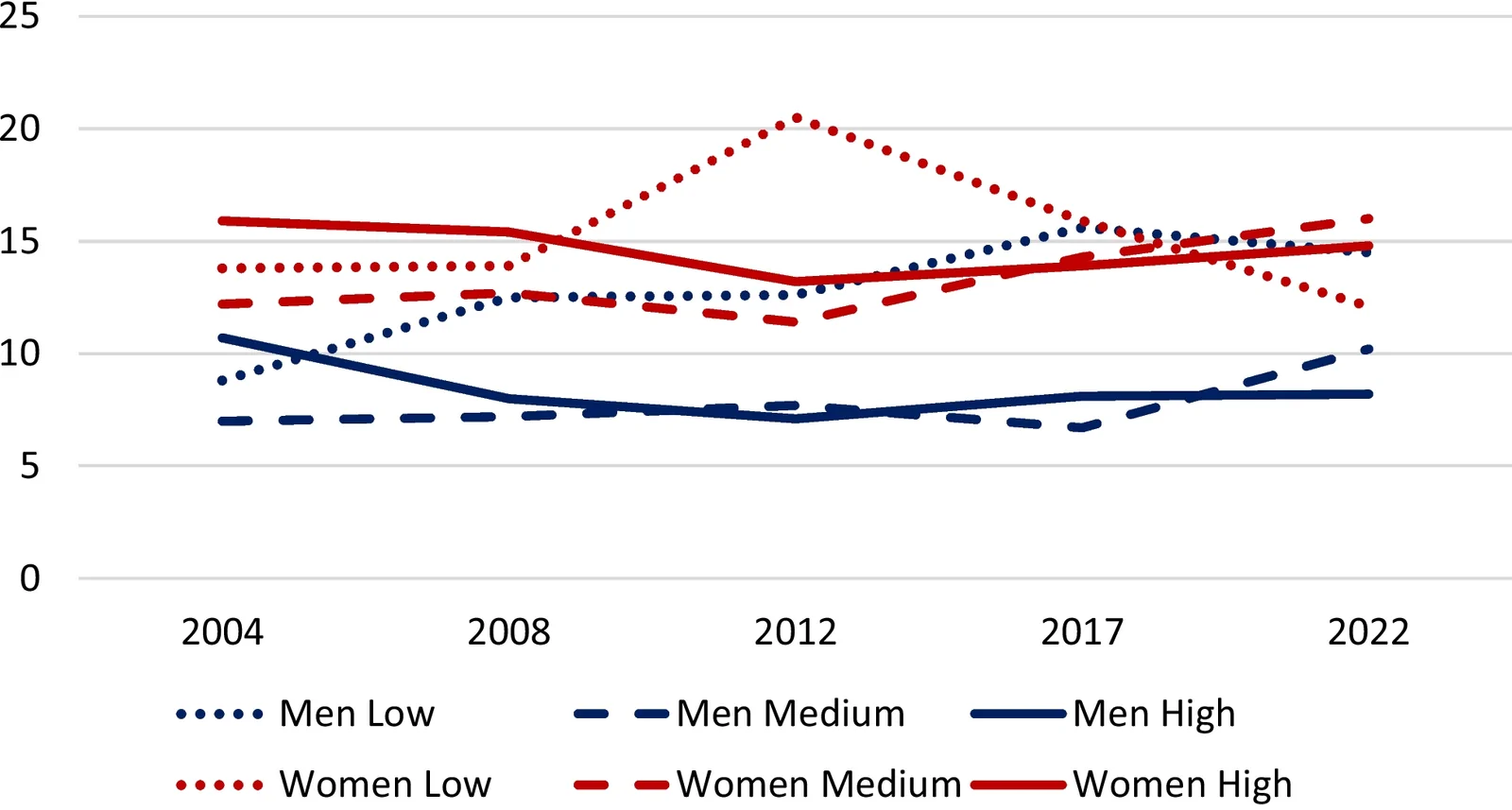
Age-standardised prevalence (%) of experiencing domestic work all or most of the time as burdensome among women and men aged 30–49 years by level of education and year

No clear trends or differences between educational levels were seen in the age-standardised prevalence of experiencing domestic work as burdensome all or most of the time in women (Fig. 2). In general, men had lower prevalence of finding domestic work burdensome than women, except for men with low education where the prevalence was similar to women from 2008 onwards.

Of note, in contrast to the younger sample, 50–69-year-old women with high education spent less time in domestic work than women with low and medium educational levels (*p* < 0.05, see figure S.1). No differences between educational levels were observed in older men. As in the younger age group, women in the older age group reported spending more time in domestic work than men did throughout the survey period.

## Discussion

In the current study of gendered trends amongst a general population of Swedish adults aged 30–49 years, women reported spending more time in domestic work than men in every survey year from 2004 to 2022. Domestic work time decreased in women whereas no trend could be seen in men. Among women, there was no difference in the prevalence of spending > 20 h/week in domestic work between educational levels. In contrast, among men, those with higher educational levels were consistently more likely to be contributing > 20 h/week to domestic work. In general, women experienced domestic work as burdensome (most or all the time) to a higher degree than men did, except for men with low education.

Although Swedish women continue to spend more time in unpaid labour than men, our results show that the gap between women and men in time in domestic work has decreased from 2004 to 2022. Whilst, on face value, this appears to align with the Swedish government’s policy target for a more even distribution of unpaid household and care work, this has been achieved *not* because men have increased their time in domestic work, but rather due to decreased time spent by women. It is not clear why Swedish women have reduced their unpaid labour time. The employment rate has been high and stable among Swedish women over the study period [19]. However, the proportion of women working part-time did decrease from about 30% to 20% during this time whereas the proportion working full-time increased correspondingly [32], which may have contributed to the declining unpaid labour time among women. Approximately two thirds of our study population had children under 18, and thus their unpaid care demands are expected to be high. Over the study period, the number of children born per woman in Sweden was highest in 2010 (approximately midway through our data collection points), and decreased thereafter [32], mainly due to decreased first child fertility [33]. Notably, despite an increase in the proportion of men taking parental benefit (from 20% in 2005 to 30% in 2018 and then levelling off) [32], no increase in time spent in domestic work was observed among men in our study. In 2007, a tax deduction for domestic services (housework and care) was introduced in Sweden [34] which could partly account for the declining trend we observed in women’s unpaid labour. Yet, whilst utility of this tax deduction has increased over time, households with high incomes purchase domestic services to a greater extent than other households and, in 2017, 69% of the tax deduction was used by households without children living at home [34].

The prevalence of spending ≥31 h per week on domestic work was consistently about double among women compared to men. This reflects the disproportionate unpaid labour burden borne by women globally [2, 35, 36]. For context, the OECD average for unpaid work is 31 h per week for women and 16 h per week for men [17]. This gendered imbalance is further reinforced by family structure patterns: in Sweden, single mothers are far more common than single fathers—about 10% versus roughly 3% among adults aged 30–49 years [37].

Notably, the results confirm our hypothesis that Swedish men (30–49 years) with higher education spend more time in unpaid work than men with lower education. This finding aligns with earlier findings from Sweden [27] and similar findings from Japan [29] and Europe [12]. However, whilst more highly educated Swedish men do greater amounts of domestic work compared to those with lower education, no actual increase in domestic work time in this group was found between 2004 and 2022*.* Conversely, a study comparing British and US dual earner coupled fathers over 30 years found that men’s time in childcare and household work increased over time and that men with higher levels of education contributed more to childcare than men with lower educational attainment [38]. However, there are large differences between countries in men’s time in unpaid labour [17]. A recent national study from South Korea found that the median time married men spent on household work, excluding childcare, was 0.1 h per day (i.e. 0.7 h per week) [35]. The corresponding time for married women was 2.6 h per day (i.e. 18.2 h per week) [35]. Finally, it is noteworthy that in the older age group of men (50–69 years) no differences between educational levels for time in domestic work were observed (see Supplementary file 1). We postulate that Swedish men in this older age group are likely to have more traditional views regarding the division of labour in the home, irrespective of educational level.

In contrast to our findings in men, we did not find any difference between educational levels in the prevalence of spending > 20 h/week in domestic work among women aged 30–49 years. This differs from the earlier results of Evertsson et al. [27], reporting that less educated women spend more time in housework than higher educated women, which in turn was true for women in the older age group 50–69 years. The finding among younger women was somewhat unexpected given higher educational status is understood to be linked to more gender egalitarian attitudes and behaviours [39] but emphasises the importance of continuous monitoring of educational trends in unpaid labour. The lack of differences observed for the women in our study may be a consequence of “spoken egalitarianism”, that is, a professed belief in gender equality that does not actually translate into correspondingly egalitarian practices, which is known to be more common in higher socioeconomic status couples [39]*.* Moreover, in Sweden, as in many other parts of the world, it is also known that ‘doing gender’ and the traditional discourse around femininity remains strong and generates conflicting influences on many women with respect to division of and time spent in unpaid labour [40].

In the present study, women experienced domestic work as burdensome most or all the time to a higher degree than men did. This is understandable due to women’s greater time in unpaid labour. However, low educated men experienced domestic work as burdensome more often than other men (and as often as women) despite their lower domestic work time, which may be attributable to an observed U-shaped association between hours spent in domestic work and burdensome domestic work [8]. In addition, there was no decreasing trend over time in experiencing domestic work as burdensome among women, even though their domestic work time decreased. Different unpaid labour tasks may entail differences in strain and have different implications for health which may contribute to this finding. For example, Ervin et al. found in the Australian longitudinal HILDA-study that household work, accounting for the greatest time spend in unpaid labour, was detrimental for both men’s and women’s mental health, whereas childcare in women and outdoor tasks in men were positively related to mental health [36]. In our study, educational differences in total unpaid labour time were studied whereas some of the previous studies have only examined housework time [27, 28, 35].

Ultimately, despite Sweden’s global renown for actively promoting more gender egalitarian societies, the findings of our study show that gender inequities persist. Moreover, our study period passes through the recent COVID-19 pandemic which shone an overdue spotlight onto unpaid labour due to the closure of schools and childcare facilities. Whilst time spent in unpaid care and domestic work increased for both men and women during the pandemic, the relative increase and intensity were much greater for women [2]. These heightened inequities further emphasised the importance of monitoring gender and socioeconomic gaps in unpaid labour, to increase their visibility [41] and to promote equality in health [42].

### Strengths and limitations

Several limitations need to be considered when interpreting the results of this study. The main limitation regards the validity and wording of the measures of unpaid work used in the surveys, and not being able to differentiate between different tasks (e.g. household work and childcare). Information on unpaid work was self-reported and therefore susceptible to recall and desirability bias which may have inflated the levels of domestic work reported. However, these possible biases are likely to be non-differential between men and women and across survey years and therefore are not expected to have impacted interpretation of the trends over time. Whilst the questions on domestic work used were part of the National Public Health Survey in Sweden 2004–2006 [43] and similar measures of domestic work have been used in other studies [5, 8, 30], a lack of validated measures regarding unpaid labour/domestic work is acknowledged [3, 15]. As such, standardised and specific questions about unpaid labour are warranted and would be immensely valuable for future research.

A further limitation is the overall survey response rate (declining from 64% in 2004 to 45% in 2022), which may have affected the representativeness of the study population. In line with previous research [44], the response rate was lower among younger persons, in men and among those with low educational level. This applies to all survey years. In 2022, the response rates were 48% in women and 41% in men, and 34% and 56% in the age groups 30–49 and 50–69 years, respectively. The response rate by educational level is not available by age group but the total response rate was 40%, 42% and 55% at low, medium and high educational levels, respectively. In addition, the proportion of persons with low educational level was relatively small, especially in the latter years, which may have affected the stability of results for low educational level. Moreover, we concede that no survey data, comparable between the survey years, on possible explanatory variables such as employment status, family status or number of children for the observed trends and differences were obtainable. As such, we were limited to using the available only sources of information on these measures from official Swedish statistics. Lastly, survey participants younger than 30 years were not included in the study since data on domestic work was not available in this age group for 2017 and since persons < 25 years may not have completed their education.

Strengths of our study include our study population, which was based on considerable random samples of the general adult population covering a large geographical area in Sweden. Whilst the study population is not a national one, the sample can be considered reasonably representative of Sweden, with the prevalence of several health indicators in line with national averages [8, 41]. Furthermore, data on gender, age and educational level were based on national registers ensuring the validity of the data. A further strength is the length of study period, where we were able to study a span of almost 20 years with uniform questions and data design, making a unique contribution to research on educational trends in unpaid labour.

## Conclusions

Our study found that whilst the gender gap in domestic work time has declined in Sweden during the last two decades, this is only due to decreases in women’s time in domestic work, and that substantial inequities in the division of unpaid labour persist. Greater efforts to implement social policies aimed at increasing men’s participation in domestic work are urgently needed, especially among men with low and middle educational levels, since it has the potential to reduce gendered health inequities. Future research should implement standardised ways to measure different dimensions and types of unpaid labour. Lastly, monitoring gender and socioeconomic disparities in unpaid labour from an international perspective is crucial for increasing visibility of this issue and promoting equality in health.

## Supplementary Information

Supplementary Material 1

## Data Availability

The data materials generated and/or analysed during the current study are not publicly available due to confidentiality and regulations under the Swedish law (Public and Privacy Act 2009:400, Chapter 24, Sect. 8), but descriptive data in table form are available from the corresponding author on reasonable request.
