# Supplementary material for: Gendered trends in unpaid labour by educational level in Sweden 2004–2022
Source: BMC Public Health. 2026 Sep 16;26:2605. doi: 10.1186/s12889-026-29473-9 (PMC13579737; doi:10.1186/s12889-026-29473-9)
Supplement: Supplementary file 1 [file 12889_2026_29473_MOESM1_ESM.docx]

**Supplementary file 1**

**Gendered trends in unpaid labour by educational level in Sweden 2004-2022 (50–69-year age group)**

Aim

As a complementary piece to our primary analysis of 30–49-year-old Swedish adults (see main manuscript), the aim of this supplementary analysis was to investigate the gendered trends in unpaid labour/domestic work by educational level in an older population sample of adults, 50–69 years, in Sweden from 2004 to 2022.

Methods

Methodology was as per our primary analysis, with study data based on a postal survey sent to a random sample of adults in 2004, 2008, 2012, 2017 and 2022. The 50–69-year age group included between 8,600 and 15,500 respondents depending on the survey year. Trends in hours spent in domestic work and in experiencing domestic work as burdensome were estimated by age-standardised proportions with corresponding 95% confidence intervals.

Results

Low educational level was more common in this age group (50–69 years), compared to those in the 30–49 age group, but as per the younger age group this decreased from 2004 to 2022 (table S.1). Not unexpectedly, only about 10% had children under 18 years living at home.

In general, the prevalence of high unpaid labour time (spending >20 hours/week in domestic work) was lower in the 50-69 age group compared to those aged 30–49 years (p<0.05). As in the younger age group, women aged 50-69 spent more time in domestic work than men did during the whole period between 2004 and 2022. Moreover, time spent in domestic work decreased in women over the study period at all educational levels, whereas no trend could be seen in men (figure S.1). In contrast to the younger cohort, 50–69-year-old women with high education spent less time in domestic work than women with low and medium educational levels (p<0.05). No differences between educational levels were observed in men.

Regarding experiencing domestic work as burdensome, no clear trends or differences between educational levels were observed (figure S.2). Women with low educational level had the highest prevalence of experiencing domestic work all or most of the time as burdensome during 2012–2022 (p<0.05).

Discussion

In line with our findings for the younger age group (30-49), Swedish women aged 50-69 years spend more time in domestic work compared to men of the same age. A decreasing trend in domestic work time was seen in these older women at all educational levels between 2004 and 2022, whereas no trend could be seen in men. In contrast to the younger age group, older women (50–69-years) with high education spent less time in domestic work than women with low and medium educational levels, while women with low education experienced domestic work burdensome to a higher degree than other women. Amongst our sample of older men (50-69-years), no differences in domestic work time or in experiencing domestic work as burdensome were observed between educational levels. Our results for men may reflect more traditional gender attitudes and behaviours in the older age group compared to those aged 30-49 years.

Table S.1. Characteristics of the study population 50-69 years by year.

| Year | 2004 | 2008 | 2012 | 2017 | 2022 |
| --- | --- | --- | --- | --- | --- |
| N | 15 485 | 15 546 | 11 820 | 8 612 | 9 981 |
| Gender (%) |  |  |  |  |  |
| Women | 51.7 | 52.2 | 53.2 | 54.9 | 54.1 |
| Men | 48.3 | 47.8 | 46.8 | 45.1 | 45.9 |
| Age group (%) |  |  |  |  |  |
| 50-59 years | 48.2 | 42.7 | 39.8 | 43.9 | 45.4 |
| 60-69 years | 51.8 | 57.3 | 60.2 | 56.1 | 54.6 |
| Educational level (%) |  |  |  |  |  |
| Low | 31.0 | 27.3 | 19.9 | 14.2 | 11.5 |
| Middle | 42.3 | 44.9 | 47.5 | 49.8 | 51.0 |
| High | 26.7 | 27.8 | 32.6 | 36.0 | 37.5 |
| Children under 18 years (%) |  |  |  |  |  |
| Yes | - | - | - | 10.1 | 9.6 |
| No | - | - | - | 89.9 | 90.4 |

|  | Men Low | Men Medium | Men High | Women Low | Women Medium | Women High |
| --- | --- | --- | --- | --- | --- | --- |
| 2004 | 8.8 | 9.7 | 9.1 | 32.2 | 29 | 22 |
| 2008 | 6.1 | 7.1 | 7.2 | 24.7 | 23.8 | 17.1 |
| 2012 | 7.3 | 8.3 | 7 | 21.5 | 16.6 | 14.8 |
| 2017 | 9.2 | 9.4 | 9 | 19.7 | 21.5 | 14.9 |
| 2022 | 8 | 8.1 | 8.7 | 15.9 | 14.8 | 11.3 |

Figure S.1 Age-standardised prevalence (%) of spending more than 20 hours per week in domestic work among women and men aged 50-69 years by level of education and year.

|  | Men Low | Men Medium | Men High | Women Low | Women Medium | Women High |
| --- | --- | --- | --- | --- | --- | --- |
| 2004 | 3.9 | 3.5 | 3.9 | 5.7 | 5.5 | 7.8 |
| 2008 | 4.3 | 4.8 | 3.2 | 6.6 | 5.8 | 6.6 |
| 2012 | 4.3 | 4.4 | 3.7 | 10.3 | 7.7 | 7.9 |
| 2017 | 4.7 | 5.4 | 3.6 | 10.9 | 7.2 | 6 |
| 2022 | 4.5 | 3.5 | 3.9 | 8.2 | 6.6 | 6.8 |

Figure S.2 Age-standardised prevalence (%) of experiencing domestic work all or most of the time as burdensome among women and men aged 50-69 years by level of education and year.
